# Ethical considerations of a randomised controlled trial of fetal intervention versus expectant management in monochorionic twin pregnancies with early‐onset selective fetal growth restriction

**DOI:** 10.1111/1471-0528.17776

**Published:** 2024-02-20

**Authors:** Richard Ashcroft, Kerry Woolfall, Smriti Prasad, Asma Khalil

**Affiliations:** ^1^ City Law School University of London London UK; ^2^ Department of Public Health, Policy and Systems Institute of Population Health and Society Liverpool UK; ^3^ Health Research Methodology The University of Liverpool Liverpool UK; ^4^ Fetal Medicine Unit, St George's Hospital St George's University of London London UK; ^5^ Fetal Medicine Unit Liverpool Women's Hospital Liverpool UK

Selective fetal growth restriction (sFGR) affects approximately 10%–15% of monochorionic twin pregnancies and is largely attributable to unequal sharing of a single placenta and the presence of unbalanced vascular anastomoses. Early‐onset sFGR (onset before 24 weeks of gestation) is relatively uncommon, but clinical management is challenging because of the risks of extreme prematurity. Selective FGR can lead to poor outcomes for both the growth‐restricted and normally grown twins, including stillbirth, neonatal death and cerebral palsy.^1^ Dilemmas in the management of these pregnancies, which may result in the loss/disability of one or both twins, has a psychological impact on the parents.^2^ In the event of demise of the smaller twin, the risks of death and neurological morbidity to the surviving co‐twin are in the order of 15% and 26%, respectively. There are three main clinical management options for early‐onset sFGR: expectant management, involving close monitoring but no active intervention; selective termination of the smaller twin, which can enable the larger twin to develop normally; and placental laser photocoagulation to separate the circulations of the twins, thus reducing the risk posed by placental anastomoses to the larger twin.^3^ Each management option has risks and ethical challenges: expectant management can be psychologically burdensome to the parents, as there is a risk that one or both twins may die; selective termination may not be acceptable to some parents; and placental laser coagulation is not only technically difficult, but of unproven effectiveness in cases of sFGR, unlike other indications like twin‐to‐twin transfusion syndrome. We have considered ethical precedents from landmark trials in the management of complicated monochorionic twin pregnancies, drawing distinctions with our context where selective termination of the smaller twin adds complex ethical dimensions to patient consent and trial feasibility.^4^, ^5^, ^6^

This situation of significant disagreement and variation in practice within the expert clinical community would seem to establish clinical equipoise, which could best be resolved through a randomised controlled trial to provide evidence‐based future practice. However, in cases of sFGR in monochorionic twin pregnancies, the research question becomes more complex because of the heterogeneity in the definition of the condition itself, which may affect the validity of a proposed trial. For example, according to the Delphi consensus, sFGR in monochorionic twin pregnancies is defined as either one solitary parameter (estimated fetal weight [EFW] of one twin <3rd centile) or at least two out of four contributory parameters (EFW of one twin <10th centile, abdominal circumference <10th centile, EFW discordance of ≥25% and umbilical artery Pulsatility Index of smaller twin >95th centile).^7^ This definition varies with professional bodies such as the International Society of Ultrasound in Obstetrics and Gynecology or the American College of Obstetricians and Gynecologists.

Clinical equipoise is usually taken to be a necessary, though not always sufficient, condition for a clinical trial to be ethically justifiable. It is further arguable that if there is no compelling reason not to perform a clinical trial, then clinical equipoise provides a compelling reason why a trial should be done, to resolve uncertainty and improve the effectiveness of treatment for patients in the future, as discussed by Ashcroft et al.^8^ The argument that there is clinical equipoise regarding interventions in early‐onset sFGR might be argued to depend on whether we consider that there is one patient (the mother) or two (or in twin pregnancies, more than two). In other words, is an unborn fetus a patient? In jurisdictions where abortion is lawful, the pragmatic answer is that the law considers the pregnant woman to be the only, or the primary, patient.^9^ However, this issue may affect the acceptability of any intervention to pregnant women, even if in general terms there is no ethical objection to selective termination or other interventions in sFGR (Mitchell et al., unpublished observations).^10^

The ethical issues relating to clinical trials in pregnancy are complex. Historically, clinical trials in women who even *might* be pregnant were not permitted, because of potential risks of teratogenesis, miscarriage or stillbirth, or preterm birth. More recently it has been recognised that this approach is unduly restrictive and undermines the development and delivery of effective care to women and their babies, whether they are pregnant or not.^11^, ^12^ However, the status of trials of treatments specifically aimed at women who are pregnant, or of treatments specifically aimed at the fetus or fetuses, remains to a degree controversial. One reason is that it is not always clear who the patient is (the mother or the fetus or both), and in some countries, it is not clear whether the fetus has moral or legal ‘personhood’, nor whose interests are being protected, as discussed by Chervenak and McCullough.^13^ In the UK, the fetus does not have any legal rights of its own, at least until it is born and has a separate existence from the mother.^14^

In practice, ethical guidance focuses on the autonomy of the woman, and her unique position as patient and pregnant woman. Whatever the theoretical basis may be for making judgements about the interests of the future child, it is arguable that only the woman herself is in a position to make those judgements, and it is clear in law and ethics that her informed consent is essential to authorising any clinical trial of a treatment during her pregnancy, regardless of whether the aim of that treatment is to benefit her, her fetus, or both.^14^ Although decision‐making during pregnancy, especially in situations where the health of the unborn fetus or fetuses is in jeopardy, can be difficult and stressful, we consider that pregnant women are fully autonomous and this must be respected. Hence, in the UK, their consent to participate in any trial remains essential.^15^

In the case of a clinical trial of management of early‐onset sFGR in monochorionic twin pregnancies, there are further ethical challenges. One is that selective reduction involves termination of pregnancy. The law in the UK does permit termination if one of several conditions is met, one of which is if ‘there is a substantial risk that if the child were born it would suffer from physical or mental abnormalities as to be seriously handicapped’ (Abortion Act 1967 s.1.1.d) [see also s.5(2)].^16^ This means that selective termination in a situation of sFGR at an early gestation is lawful in most cases. However, the legality and public acceptability of termination of pregnancy, even on medical grounds such as the health of the pregnant woman or the viability of the fetus, vary considerably between jurisdictions. Therefore, the permissibility of fetal reduction varies, and it may not always be possible to consider it as an intervention in sFGR. In the UK this approach is lawful, and we argue that the state of clinical equipoise relating to the safest, most effective and most acceptable intervention in early‐onset sFGR extends to include selective termination of pregnancy in that context. Nevertheless, termination of pregnancy is controversial, and would not be acceptable to all women, even in these circumstances.

In the context of a randomised trial, where selective termination of one fetus is one of the treatment arms, a two‐stage consent is required: consent to being randomised, and consent to undergoing selective termination, should that be the treatment arm to which the patient is randomised. Alternatively, a trial design where patients are ‘pre‐randomised’, and consent only to the specific treatment they receive and to data collection may be considered.^17^, ^18^, ^19^, ^20^, ^21^ However, pre‐randomised designs pose the problem that the patient needs to know that they are being randomised without their knowledge or consent, offered participation in a trial, and that other participants in the trial are being offered alternative treatments, before or after randomisation. This takes us to the second ethical issue: patients may have legitimate moral preferences for one treatment or the other, independently of the question of clinical equipoise as to the safety and effectiveness of the different management options. A simple randomisation between options requires both clinical equipoise on the part of the clinical community and acceptance on the part of patients that such clinical equipoise exists, and moral equipoise or indifference as to the moral legitimacy of the management options. This condition of moral equipoise or indifference may be difficult to meet. Again, one solution may be a pre‐randomised design, so that the patient has only to consent or refuse consent to the specific option they are offered, and their moral attitude towards it is then part of that consent process. However, this does not solve the problem mentioned above, that they may have a moral preference for a treatment option they are not being offered, but other patients are. Pre‐randomisation in trials can help to counter contamination bias. The caveat remains that if most patients opt for pre‐randomisation to a particular treatment arm and refuse consent for another, it can be problematic, and the trial may not be feasible. This is a standard issue in pre‐randomised designs that may be mitigated by perhaps an ‘intention‐to‐treat’ analysis.

This may be more challenging in the context of early‐onset sFGR, where there may be a higher than anticipated decline rate in allocated treatment options, affecting the intention‐to‐treat analysis. Some of the potential strategies to enhance patient acceptance of allocated treatments and maintain the integrity of the intention‐to‐treat principle would include providing comprehensive counselling before consent to ensure informed choices. In pre‐randomised designs, although patients are initially allocated to one arm, they maintain the right to withdraw and receive other available standard treatments.

One way out of this conundrum is empirical: assuming that only a minority of patients in this situation have strict and categorical objections to termination of pregnancy, for most patients their decision about what to do will involve some deliberation about the options. The trial design may be ethical in and of itself, but the moral question about whether to accept a termination of pregnancy (or an alternative management strategy that may lead to death or disability of one or both fetuses) can only be answered by each patient for themselves. That being so, there is no simple answer to the question of whether such a trial is ethical or not. It is definitely lawful, but it may or may not be acceptable. This can be determined by empirical research, principally with women who are either going through a monochorionic twin pregnancy complicated by early sFGR (as to whether they would consent to participate in such a trial, and if so, what their reasoning would be), or who have been through such a pregnancy (retrospectively, would they have wanted this option and how would they have chosen if that had been possible). These two categories of women are best placed to help establish how potential trial participants would make decisions about trial participation, and have the clearest understanding of the ethical, moral and psychological issues at stake.

This highlights another issue, which is the psychological burden of choice. In one scenario (expectant management with no other treatment being offered), women may experience the psychological stress of ‘waiting and seeing’, knowing that there is a high probability of an adverse outcome to their pregnancy. In the active treatment scenarios, this psychological stress may be accompanied by the stress of having to make a positive choice and feel the responsibility for the outcome which may go with that. Participation in a trial may also have a psychological benefit, in terms of feeling that one is doing something that may help others in the future, as well as hoping to improve outcomes in their own pregnancy. Nevertheless, such decision‐making may also have a psychological burden, in terms of both choosing to participate in a trial and having no direct say in what treatment is then delivered.

Additionally, in some jurisdictions where health care is available only on a paid basis, or other barriers to treatment may exist, there is an ethical risk that some patients may participate in a trial because this is the only way they can access the treatment they prefer. This presents a question of justice. However, in the UK, this is rarely an issue for patients accessing treatment through the National Health Service.

In summary, clinical equipoise between the different management options for early‐onset sFGR in monochorionic twin pregnancies and the need for evidence‐based clinical practice provide a strong justification for a clinical trial. However, the moral complexities of selective termination and the psychological burden of choice mean that greater than usual weight is placed on the importance of informed consent. Empirical research is needed to explore the moral issues that patients consider when consenting to clinical trials. This research may use the Four Principles approach as the analytical framework and draw on the Declaration of Helsinki for international ethical standards, determining what makes a trial acceptable to patients and feasible to conduct.^22^, ^23^ Specifically, this aspect of our research is being addressed in Work Package 2 (WP2) of the FERN (Intervention or Expectant Management for Early Onset Selective Fetal Growth Restriction in Monochorionic Twin Pregnancy) study.^24^ WP2 is dedicated to understanding the factors that influence potential trial participants' willingness to engage in randomised trials, especially in the context of interventions that include ethically challenging options such as selective termination of the smaller fetus.

## AUTHOR CONTRIBUTIONS

RA and AK conceptualised the article. RA wrote the first draft. AK, KW and SP critically reviewed the first draft and provided inputs. All authors have approved the final manuscript.

## FUNDING INFORMATION

This work is funded by National Institute for Health and Care Research (NIHR) via grant number NIHR128596.

## CONFLICT OF INTEREST STATEMENT

The authors declare no conflicts of interest.

## ETHICS STATEMENT

No ethical approval was deemed necessary for this work.

## Data Availability

Not applicable as no data were generated in this study.

## References

[1] Choi ES , Jung YM , Cho KD , Ha S , Sohn J , Hong SJ , et al. Long‐term adverse neurodevelopmental outcomes of discordant twins delivered at term: a nationwide population‐based study. BJOG. 2023;130:1370–1378. 10.1111/1471-0528.17494 37077036

[2] Khalil A , Thilaganathan B . Selective fetal growth restriction in monochorionic twin pregnancy: a dilemma for clinicians and a challenge for researchers. Ultrasound Obstet Gynecol. 2019;53(1):23–25. 30125419 10.1002/uog.20093

[3] Colmant C , Lapillonne A , Stirnemann J , Belaroussi I , Leroy‐Terquem E , Kermovant‐Duchemin E , et al. Impact of different prenatal management strategies in short‐ and long‐term outcomes in monochorionic twin pregnancies with selective intrauterine growth restriction and abnormal flow velocity waveforms in the umbilical artery Doppler: a retrospective observational study of 108 cases. BJOG. 2021;128(2):401–409. 32416618 10.1111/1471-0528.16318

[4] Stirnemann J , Slaghekke F , Khalek N , Winer N , Johnson A , Lewi L , et al. Intrauterine fetoscopic laser surgery versus expectant management in stage 1 twin‐to‐twin transfusion syndrome: an international randomized trial. Am J Obstet Gynecol. 2021;224(5):528.e1–528.e12. 10.1016/j.ajog.2020.11.031 33248135

[5] Quintero R , Kontopoulos E , Williams ME , Sloop J , Vanderbilt D , Chmait RH . Neurodevelopmental outcome of monochorionic twins with selective intrauterine growth restriction (SIUGR) type II: laser versus expectant management. J Matern Fetal Neonatal Med. 2021;34(10):1513–1521. 31309857 10.1080/14767058.2019.1638902

[6] Senat MV , Deprest J , Boulvain M , Paupe A , Winer N , Ville Y . Endoscopic laser surgery versus serial amnioreduction for severe twin‐to‐twin transfusion syndrome. N Engl J Med. 2004;351(2):136–144. 15238624 10.1056/NEJMoa032597

[7] Khalil A , Beune I , Hecher K , Wynia K , Ganzevoort W , Reed K , et al. Consensus definition and essential reporting parameters of selective fetal growth restriction in twin pregnancy: a Delphi procedure. Ultrasound Obstet Gynecol. 2019;53(1):47–54. 29363848 10.1002/uog.19013

[8] Ashcroft R . Equipoise, knowledge and ethics in clinical research and practice. Bioethics. 1999;13(3–4):314–326. 11657242 10.1111/1467-8519.00160

[9] Ashcroft R . Ethical issues in a trial of maternal gene transfer to improve Foetal growth. In: Baylis F , Ballantyne A , editors. Clinical research involving pregnant women [Internet]. Cham: Springer International Publishing; 2016 [cited 2023 Aug 3]. p. 247–263. (Research Ethics Forum). 10.1007/978-3-319-26512-4_14

[10] Sheppard M , Spencer RN , Ashcroft R , David AL , EVERREST Consortium . Ethics and social acceptability of a proposed clinical trial using maternal gene therapy to treat severe early‐onset fetal growth restriction. Ultrasound Obstet Gynecol. 2016;47(4):484–491. 26968870 10.1002/uog.15880

[11] Macklin R . Enrolling pregnant women in biomedical research. Lancet. 2010;375(9715):632–633. 20198725 10.1016/s0140-6736(10)60257-7

[12] Vousden N , Haynes R , Findlay S , Horby P , Landray M , Chappell L , et al. Facilitating participation in clinical trials during pregnancy. BMJ. 2023;380:e071278. 36746514 10.1136/bmj-2022-071278

[13] Chervenak FA , McCullough LB . Ethical dimensions of the fetus as a patient. Best Pract Res Clin Obstet Gynaecol. 2017;43:2–9. 28190695 10.1016/j.bpobgyn.2016.12.007

[14] Cao KX , Booth A , Ourselin S , David AL , Ashcroft R . The legal frameworks that govern fetal surgery in the United Kingdom, European Union, and the United States. Prenat Diagn. 2018;38(7):475–481. 29663461 10.1002/pd.5267 PMC6033164

[15] Lupton MGF , Williams DJ . The ethics of research on pregnant women: is maternal consent sufficient? BJOG. 2004;111(12):1307–1312. 15663112 10.1111/j.1471-0528.2004.00342.x

[16] Participation E . Abortion Act 1967 [cited 2023 Jan 20]. Available from: https://www.legislation.gov.uk/ukpga/1967/87/section/1

[17] Zelen M . A new design for randomized clinical trials. N Engl J Med. 1979;300(22):1242–1245. 431682 10.1056/NEJM197905313002203

[18] Zelen M . Randomized consent designs for clinical trials: an update. Stat Med. 1990;9(6):645–656. 2218168 10.1002/sim.4780090611

[19] Schellings R , Kessels AG , ter Riet G , Sturmans F , Widdershoven GA , Knottnerus JA . Indications and requirements for the use of prerandomization. J Clin Epidemiol. 2009;62(4):393–399. 19056237 10.1016/j.jclinepi.2008.07.010

[20] Homer CSE , Davis GK , Brodie PM , Sheehan A , Barclay LM , Wills J , et al. Collaboration in maternity care: a randomised controlled trial comparing community‐based continuity of care with standard hospital care. Br J Obstet Gynaecol. 2001;108(1):16–22. 10.1111/j.1471-0528.2001.00022.x 11212998

[21] Hatcher S , Sharon C , House A , Collins N , Collings S , Pillai A . The ACCESS study: Zelen randomised controlled trial of a package of care for people presenting to hospital after self‐harm. Br J Psychiatry. 2015;206(3):229–236. 25614531 10.1192/bjp.bp.113.135780

[22] Beauchamp TL , Childress JF . Principles of biomedical ethics. 8th ed. New York, NY: Oxford University Press; 2019.

[23] World Medical Association . World Medical Association Declaration of Helsinki: ethical principles for medical research involving human subjects. JAMA. 2013;310(20):2191–2194. 24141714 10.1001/jama.2013.281053

[24] FERN . Intervention or expectant management for early onset selective fetal growth restriction in monochorionic twin pregnancy – NIHR Funding and Awards [Internet]. [cited 2023 Dec 9]. Available from: https://fundingawards.nihr.ac.uk/award/NIHR128596

